# Multi-Class Wound Classification via High and Low-Frequency Guidance Network

**DOI:** 10.3390/bioengineering10121385

**Published:** 2023-12-01

**Authors:** Xiuwen Guo, Weichao Yi, Liquan Dong, Lingqin Kong, Ming Liu, Yuejin Zhao, Mei Hui, Xuhong Chu

**Affiliations:** 1 School of Optics and Photonics, Beijing Institute of Technology, Beijing 100081, China; 3120235140@bit.edu.cn (X.G.); 3120215346@bit.edu.cn (W.Y.); konglingqin3025@bit.edu.cn (L.K.); bit411liu@bit.edu.cn (M.L.); yjzhao@bit.edu.cn (Y.Z.); huim@bit.edu.cn (M.H.); chuxuhong001@bit.edu.cn (X.C.); 2 Beijing Key Laboratory for Precision Optoelectronic Measurement Instrument and Technology, Beijing 100081, China; 3 Yangtze Delta Region Academy of Beijing Institute of Technology, Jiaxing 314019, China

**Keywords:** multi-class wound classification, high and low-frequency information, deep learning, two-branch network, transfer learning

## Abstract

Wound image classification is a crucial preprocessing step to many intelligent medical systems, e.g., online diagnosis and smart medical. Recently, Convolutional Neural Network (CNN) has been widely applied to the classification of wound images and obtained promising performance to some extent. Unfortunately, it is still challenging to classify multiple wound types due to the complexity and variety of wound images. Existing CNNs usually extract high- and low-frequency features at the same convolutional layer, which inevitably causes information loss and further affects the accuracy of classification. To this end, we propose a novel High and Low-frequency Guidance Network (HLG-Net) for multi-class wound classification. To be specific, HLG-Net contains two branches: High-Frequency Network (HF-Net) and Low-Frequency Network (LF-Net). We employ pre-trained models ResNet and Res2Net as the feature backbone of the HF-Net, which makes the network capture the high-frequency details and texture information of wound images. To extract much low-frequency information, we utilize a Multi-Stream Dilation Convolution Residual Block (MSDCRB) as the backbone of the LF-Net. Moreover, a fusion module is proposed to fully explore informative features at the end of these two separate feature extraction branches, and obtain the final classification result. Extensive experiments demonstrate that HLG-Net can achieve maximum accuracy of 98.00%, 92.11%, and 82.61% in two-class, three-class, and four-class wound image classifications, respectively, which outperforms the previous state-of-the-art methods.

## 1. Introduction

Trauma is not only physically harmful to the patient, but there are considerable costs for diagnosing the trauma [1]. According to the latest data from the World Health Organization, approximately 180 million people worldwide are hospitalized each year for injuries, including diabetic foot ulcers [2,3,4], venous ulcers [5,6], pressure ulcers [7], and surgical wounds. Failure to diagnose wounds in time can lead to infection, sepsis, amputation, and even death. Traditional assessment of wound type relies heavily on the visual examination of wound characteristics by the clinician, and the diagnosis mainly depends on the experience level of the clinician, with low accuracy [8]. Generally, accurate diagnosis of wound types requires various examinations, but hospitals around the world do not necessarily have sufficient medical resources. Simultaneously, the shortage of surgeons in some areas can also prevent patients from receiving timely and high-quality wound care [9]. Therefore, low-cost, fast and accurate classification based on wound images has become an important task in smart medical care. The field of wound image classification currently faces several challenges, including the lack of diverse and well-annotated datasets, class imbalance, variable wound appearance, and difficulty in maintaining model robustness for clinical applications. These challenges impact the development of accurate and reliable deep learning models. Accurate wound classification can improve the quality of patient wound care, reduce medical costs through targeted intervention, and enhance the overall efficiency of medical services, with far-reaching potential implications for diagnosis and treatment. By continuously optimizing intelligent wound classification, individualized treatments can be quickly formulated for different wound patients in the future.

With the increasing availability of datasets, deep learning models have made rapid advancements in the field of medical imaging [10,11,12,13]. Since the remarkable success of the AlexNet architecture in the ImageNet competition, the potential of deep learning for image processing has been widely recognized [14]. Deep learning models like AlexNet [15], VGGNet [15] and ResNet [16] are already applied for wound image classification. The ResNet introduces residual blocks through skip connections, making the network handle deeper layers without suffering from performance degradation. For feature extraction, skip connections allow the combination of different convolution operators. Later, several improvement models based on ResNet appeared, such as Res2Net [17], ResNeSt [18], IResNet [19], and so on. Res2Net introduces a hierarchical structure within a residual block, replacing the traditional 3 × 3 convolutional kernel, which is the multi-scale feature representation. This technique enhances the perception of each network layer and enables a finer representation of multi-scale features.

Currently, deep learning has been extensively applied in various fields of wound image analysis [20,21,22,23,24,25]. In a study conduct in 2017 [26], the performance of computers for skin cancer classification is compared to manual classification by dermatologists. The results show that Artificial Intelligence (AI) is able to classify skin cancer with an accuracy similar to dermatologists. Rostami and Niezgoda [27] utilize the pre-trained deep convolutional neural network, AlexNet, to classify burn wound images into two to three distinct categories. Comparison with previous work using the same dataset shows that the classification accuracy of the classifier proposed in this paper is improved by more than 8%. Goyal et al. [28] use CNN in Diabetic Foot Ulcers (DFU) classification for the first time. They propose the DFUNet architecture to recognize the feature differences between healthy skin and DFU. DFUNet incorporates two types of convolutional layers, a traditional convolutional layer that uses a single convolutional filter at the beginning of the network, and a parallel convolutional layer that uses multiple convolutional layers to extract multiple features from the same input. Finally, the AUC score for DFUNet is 0.961 by 10-fold cross-validation. Although deep learning has been successful in the field of wound images, multi-class wound classification based on limited wound image datasets is still a challenge. In recent years, several studies have proposed methods to classify multiple types of wounds. Rostami et al. [29] develop a CNN-based ensemble classifier to classify wound images into surgical ulcers, diabetic ulcers, and venous ulcers. The research presents a novel wound image dataset consisting of 538 images of four different types of wounds. They originally design patch classifiers with a fine-tuned AlexNet architecture to categorize wound patches into different wound types. In the image classification task, for each input image, a feature vector is first created based on the designed patch classifier and another AlexNet trained on the whole image. The feature vectors are then fed into the MLP to obtain an ensemble image classifier. For the binary classification, they achieve a maximum classification accuracy of 96.4% and an average classification accuracy of 94.28%. For the three-class wound classification, they get 91.9% and 87.7% classification accuracy. Anisuzzaman et al. [30] develop a multimodal classifier (WMC) based on deep neural networks to classify wound images into various categories, including diabetes, pressure ulcers, surgical wounds, and venous ulcers, based on the corresponding location of the wounds. This is the first multimodal network to use images and locations for wound classification. To prepare the location data, they also develop a body map to help clinicians recording the wound location. The WMC network is composed of two networks, the wound image classifier (WIC) and the wound location classifier (WMC). The WIC network is built based on VGG16, VGG19, ResNet50 and InceptionV3 transfer learning models. WLC networks can classify wound locations using multilayer perceptron (MLP) or long short-term memory (LSTM) networks. Across all classification experiments, the highest accuracy in wound classification ranging from 72.95% to 98.08%.

In view of current medical needs and limitations of existing work, the motivations for our research can be listed as follows:

- Wound classification is an important task in the field of medical image processing. By fully exploring the potential of deep learning in medical image processing, our research is crucial for correctly and quickly classifying different types of wounds, helping doctors develop appropriate treatment plans and improve treatment effects.
- Wound images contain abundant high- and low-frequency information, which is an important basis for diagnosing wound types. The high-frequency part usually contains the details and texture of the image, while the low-frequency part reflects more of the overall structural characteristics such as wound size, shape, and location. By processing high- and low-frequency information separately, it is expected to better capture the details and overall characteristics of wound images and improve the accuracy of classification.
- The use of a single network in previous studies may be difficult to capture information at different scales at the same time. We adopt a dual-branch structure that can effectively fuse features at different scales, including high- and low-frequency information, to more comprehensively capture the details and overall structure of the wound image [31]. This helps cope with diversity wounds and improves the generalization performance of the network.
- Wound images are often interfered by factors such as illumination, occlusion, and noise, which affect the performance of traditional methods. Our work aims to improve the adaptability to complex images by processing high- and low-frequency information separately, thereby improving the robustness of wound classification.

In summary, our contributions are as follows:

- We propose a novel HLG-Net for multi-class wound classification, and it contains two main branches: High-Frequency Network (HF-Net) and Low-Frequency Network (LF-Net), where the HF-Net focuses on high-frequency information extraction, while the LF-Net can explore low-frequency information of the image. Extensive experiments demonstrate that our HLG-Net can outperform other state-of-the-art methods on multi-class wound classification performance.
- We utilize pre-trained models ResNet and Res2Net as the backbones of the HF-Net, respectively. Both ResNet and Res2Net can allow the network to capture more complex and informative features that contain high-frequency details and texture information. Moreover, feature attention module and enhancing block are employed to improve the representation capability of HF-Net and multi-scale feature extraction, respectively.
- In LF-Net, we adopt a novel Multi-Stream Dilated Convolutional Residual Block (MSDCRB) as the core of feature extraction. A larger receptive field introduced by multi-stream dilation convolution layers can capture global information of wound images, thus pushing HLG-Net to achieve a better classification performance.
- To take full advantage of feature streams from different branches HF-Net and LF-Net, we propose a fusion module to fuse them together and output the final classification result by softmax layer with 4 output channels. The features extracted from each branch are flattened and concatenated to create the final feature vector, which is fed into the multilayer perceptron for wound classification.

## 2. Materials and Methods

### 2.1. Transfer Learning

To classify wound images, it is difficult to train a deep convolutional neural network from scratch. CNN requires a large amount of training data, which is difficult to obtain in the medical field. The collection of multiple images may involve many people, resulting in inconsistent image standards. Furthermore, training the network from the beginning requires repeatedly adjusting the weights and hyperparameters of the network, which is time-consuming [32].

Pre-trained models have been successfully applied to various computer vision tasks, with pretrained networks for image classification typically performed on the ImageNet dataset. The ImageNet dataset consists of 14,197,122 images, including 1000 classes such as animals, plants, and cars [33]. Many deep CNNs trained on natural images are not customized for a specific dataset or task. With increasing differences between the pre-training dataset and the target dataset, the network features become less transferable. However, even transferring features from obviously different tasks outperforms random initial weights [34]. Transfer learning can accelerate network convergence, reduce computational requirements and improve network performance [35].

### 2.2. Proposed Architecture

To address the complexity of wound images and the overfitting problem associated with small wound dataset, we propose the High and Low-frequency Guidance Network (HLG-Net) for multi-class wound classification. The low-frequency information corresponds to the overall shape of the wound, while the high-frequency information corresponds to the details of the wound. The combination of high and low-frequency information from wound images provides a more comprehensive wound characterization, which is essential for wound classification [36].

**Figure 1:**
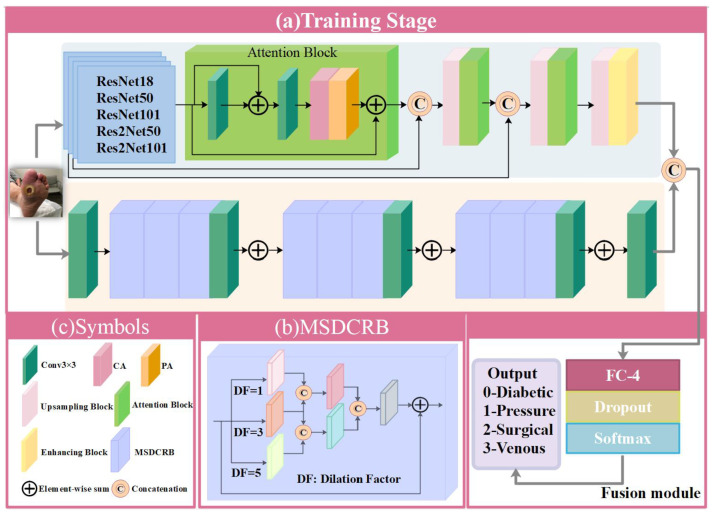
Illustration of the HLG-Net model architecture.

Based on the above-mentioned problem of wound classification and the analysis of high-frequency and low-frequency information of wound images, HLG-Net is a two-branch network, and its structure is shown in Figure 1a. Specifically, the HLG-Net contains two branches: High-Frequency Network (HF-Net) and Low-Frequency Network (LF-Net). The former can extract high-frequency information from the input image, while the latter focuses on low-frequency information extraction. When the wound image has more low-frequency information, the classification effect of HF-Net will be poor [31]. LF-Net can be used as a supplement to extract the low-frequency information. The stacked layers and residual blocks of ResNet [16] allow the network to learn more complex and abstract features which can effectively capture the high-frequency details and texture information of wound images. Besides Res2Net [17] increases the perception of features at different scales, allowing the network to better capture the detail and texture information of wound images. So we employ pre-trained ResNet and Res2Net as the backbone of the HF-Net, respectively. We introduce feature attention module after the Res2Net convolutional layer in the HF-Net. Previous CNN-based wound classification networks treat channel and pixel features equally, but the distribution of wounds in wound images is not uniform. Therefore, we use the module to make the network more focus on the wound color fidelity. In addition, we add the pixelshuffle upsampling module [37], and the enhancing block [38] to capture information at different scales. We create the LF-Net to focus on low-frequency and global information of wound images. The operation of Dilated Convolution (DC) can increase the receptive field of the convolutional layer without increasing the number of parameters, which helps to capture global information in the wound images [39]. We use the Multi-Stream Dilation Convolution Residual Block (MSDCRB) to maintain the important features from different scales for dealing with complex wound images and preserve the image details [40]. Finally, the fusion module combines the high and low-frequency features from the two branches. In general, our proposed HLG-Net contains two main branches, HF-Net and LF-Net, which respectively focus on learning different frequency domain information in wound images. When the wound image contains more low-frequency information, LF-Net can be used as a supplement to HF-Net. The two network branches work together organically to improve the classification effect. At the end of the network, through the fusion module, the output features of the two branches are concatenated in the channel dimension to form a more representative feature vector, which provides support for comprehensive wound feature extraction and classification.

#### 2.2.1. HF-Net

High-frequency information can provide local details of the wound, which is essential for the classification for different wounds. ResNet [16] is commonly used in image processing tasks to fully extract the high-frequency information of images. ResNet introduces residual connections, which can alleviate the problem of gradient disappearance and obtain a deeper network structure. The stacked layers and residual blocks of ResNet enable the network to learn more complex and abstract features, which can effectively capture high-frequency details and texture information of wound images. ResNet can deliver the original features directly to the later feature layers through residual connections, which also contributes to retain the high-frequency details of the image and avoid losing information. Res2Net is an improved model based on ResNet, which uses a multi-scale branch structure to increase the receptive field of the network, allowing the network to better capture multi-scale features. Res2Net [17] expands a normal convolutional layer into multiple parallel sub-branches. This approach increases the perception of features at different scales, enabling the network to better capture the details information of wound images. Therefore, we use pretrained ResNet18, ResNet50, ResNet101, Res2Net50 and Res2Net101 as the backbone of HF-Net, respectively.

**Figure 2:**
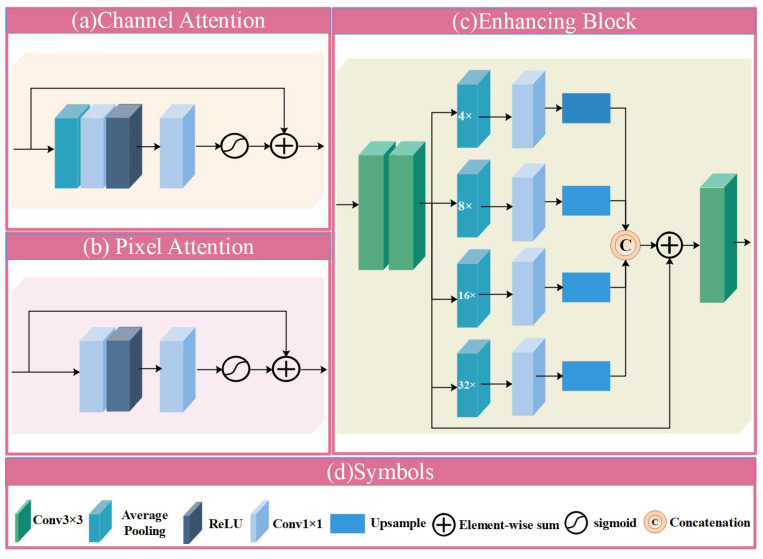
The structure of modules in HLG-Net.

Moreover, pre-trained models can reduce the overfitting problem caused by the limited wound images [41]. We employ pre-trained ResNet and Res2Net, discarding fully connected layers [42]. As shown in Figure 1a, we introduce the feature attention module after the Res2Net convolutional layer in HF-Net, which consists of multiple local residual connections and the feature attention [43]. The multiple local residual connections allow the HF-Net to focus on more high-frequency information from wound images. The structure also preserves the information from shallow layers and propagates it to deeper layers. Different channel features contain different weights, and the distribution of wounds exhibits inhomogeneity on different pixels of the image. As shown in Figure 2a,b, we employ feature attention that includes Channel Attention (CA) with Pixel Attention (PA) [44]. The CA can assign more weight to important features, allowing different features to adaptively learn distinct weight. The PA treats different pixels unequally. Previous CNN-based wound classification networks treat channel and pixel features equally, but the distribution of wounds is not uniform. The feature attention module allows the network to pay more attention to valid information such as wound color fidelity. In addition, we adopt the pixelshuffle upsampling module [37], and the enhancing block [38]. The size of the wound area in wound image is different, therefore we employ an enhancing block to capture information at different scales. As illustrated in Figure 2c, the enhancing block consists of two front-end convolutional layers with a kernel size of 3 × 3. The outputs of the front-end convolutional layers are downsampled in an average pooling operation with kernel sizes of 4 × 4, 8 × 8, 16 × 16, and 32 × 32 to generate a four-scale pyramid [45]. Convolution with the kernel size of 3 × 3 is used to concatenate the feature maps for obtaining feature details at different scales. Most of the low-frequency information of the wound image is lost due to the characteristics of the high-frequency network constituent modules. Therefore, the second branch that can effectively explore low-frequency information is essential.

#### 2.2.2. LF-Net

In order to better utilize the limited wound image information, we create a second branch to focus on the low-frequency and global information of wound images [15]. The LF-Net consists of multiple convolution layers with kernel size 3 × 3, MSDCRB and residual connections. Note that this branch only uses the wound image dataset, without any dataset for pre-training. Dilated Convolution (DC) expands the receptive field of the convolution layer by introducing dilation rate [39]. It can be used to increase the receptive field of a convolution operation to better capture contextual information. A dilation rate of 1 is equivalent to a normal convolution operation. when the dilation rate is greater than 1, the receptive field of the convolution kernel is increased accordingly. The operation of DC can increase the receptive field of the convolutional layer without increasing the number of parameters, which helps to capture the global information in the wound images. We propose MSDCRB to capture important features at different wound image pyramid levels. As shown in Figure 1b, MSDCRB consists of Multi-Stream Dilation Convolution (MSDC) [40] module with residual connection. Residual connections allow the network to better capture and process deeper features, thus improving the representation of the network. ${Conv}_{3\times 3}$ denotes the convolution operation with the kernel size of 3 × 3. This operation is to convert image space into feature space.

Mathematically, *MSDCRB* can be described as follows:

(1) $$D_{r}={\mathrm{Conv}}_{r}\left(I\right),r=1,3,5$$

where *I* is the input feature and $D_{r}$ is corresponding output, respectively. *r* is the dilation factor based on the convolution with the kernel size of 3 × 3.

(2) $$D_{k,j}=\sigma \left({\mathrm{Conv}}_{1\times 1}\left(C\left[D_{k},D_{j}\right]\right)\right)$$

where *C* is the concatenation operation and ${Conv}_{1\times 1}$ is the convolution operation with kernel size of 1 × 1. σ is the ReLU activation function and $D_{k,j}$ is the fuse output.

(3) $$MSDC={\mathrm{Conv}}_{1\times 1}\left(C\left[D_{1,3},D_{3,5}\right]\right)$$

where MSDC is the output of Multi-Stream Dilation Convolution.

(4) MSDCRB=I+MSDC

where MSDCRB is the sum of the input feature *I* and the output of MSDC.

MSDCRBs can maintain the important features from different scales to deal with complex wound images and preserve the image details. LF-Net always maintains the original resolution of the input image and does not use any downsampling operations. This operation avoids losing the fine features of the wound image and can extract more low-frequency information of wound images, which can be used as a supplement to HF-Net. Since it can extract more detailed features of wound images, it is more prone to overfitting problems. Therefore, using transfer learning in the first branch is necessary to reduce overfitting.

#### 2.2.3. Fusion Module

At the end of the HLG-Net, we design a fusion module that connects different output features of the two branches. We first concatenate the channel dimensions of the two branches for feature fusion, and then use a fully connected layer and a softmax layer with 4 output channels. For different classification tasks, the output layer uses a softmax layer or a sigmoid layer to achieve multiple or binary classification of wounds, respectively. The reason for using only one fully connected operation is that both branches have already sufficiently learned the distinctive features of wound images. The extracted features from each sub-network are flattened and concatenated to create the final feature vector, which is fed into the multilayer perceptron for wound image classification. Subsequently, the trained model is evaluated on test images to evaluate the performance of the method [46]. The different wound image features learned by each CNN architecture are combined to generate the final feature vector.

### 2.3. Loss Function

The loss function is a cross-entropy loss function, which is used to solve multi-classification problems and can also be used to solve binary classification problems [15]. The formula is as follows:

(5) $$\begin{array}{cc} loss(x,class) & =-log\left(\frac{exp(x[class])}{\sum_{i}exp\left(x\left[i\right]\right)}\right)\\ & =-x\left[class\right]+log\left(\sum_{i}exp\left(x\left[i\right]\right)\right) \end{array}$$

where *x* is the input and also the output of the last layer of the network. class denotes the number of categories. *i* denotes the index value corresponding to each class.

In the multi-classification task of images, the cross-entropy loss function can be used to measure the difference between the predicted probabilities of the model and the true labels. Generally, in multi-classification tasks, the last layer uses the softmax function as an activation function to convert the model output into a probability distribution for each category. The cross-entropy loss function calculates the cross-entropy between the predicted probability distribution and the true label as the optimization target for training the network.

## 3. Experiment

### 3.1. Dataset: AZH Wound and Vascular Center Database

**Figure 3:**
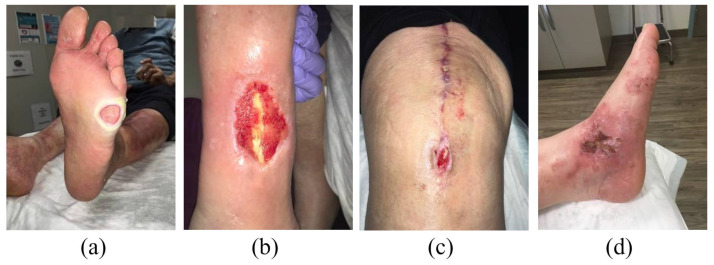
Wound samples from the AHZ dataset. (**a**) Diabetic foot ulcer wound image. (**b**) Pressure ulcer wound image. (**c**) Surgical wound image. (**d**) Venous ulcer wound image.

In this study, we utilize an open-source dataset derived from the work of Rostami et al. [29]. The wound dataset was collected over a two-year period at the AZH Wound and Vascular Center in Milwaukee, Wisconsin, a specialized wound and vascular care facility, and the wound images in the dataset were derived from real medical situations. There are a total of 538 images representing four different types of wounds: Diabetic wounds, Pressure wounds, Surgical wounds, and Venous wounds, with the abbreviations shown in the Table 1. Wound images were taken with an iPad Pro (software version 13.4.1) and a Canon SX 620 HS digital camera and annotated by wound specialists from the AZH Wound Center. The image width is 320 to 700 pixels and the height is 240 to 525 pixels. There are no specific environmental or lighting conditions during image acquisition process. Figure 3 illustrates wound image samples from the AHZ dataset. The dataset is divided into a 70% training set, a 20% validation set, and a 10% test set. The specific partitioning is presented in Table 2. Due to training and testing on a realistic open-source dataset, our model is able to recognize and classify various types of real wounds. This demonstrates the clinical reliability and utility of our wound classification model.

### 3.2. Data Preprocessing

Given the privacy of patients, publicly available datasets containing images of multiple types of wounds are scarce. Moreover, it is also challenging to access large datasets through healthcare institutions. Therefore, we use a limited wound dataset. The limited number of input samples increases the risk of overfitting during CNN training, resulting in a decrease in classification accuracy [47]. Data preprocessing can partially solve the problems of lack of data sets and imbalanced distribution of different types of wound images. Data augmentation is widely used in data preprocessing to generate additional training data from existing training sets. Traditional image enhancement methods include geometric transformations such as rotation, flipping, and random scaling, as well as color transformations [48].

Another preprocessing method is image normalization [49], which aims to obtain the same range of values for each input image before feeding it into the CNN model, thereby helping to improve the convergence speed of the model. The input images are normalized to a standard normal distribution with a range of [0,1]. The normalization process is calculated as follows:

(6) $$x_{N}=\frac{x-x_{min}}{x_{max}-x_{min}}$$

where *x* is the pixel intensity. $x_{min}$ and $x_{max}$ correspond to the minimum and maximum intensity values of the input image, respectively.

Region of Interest (ROI) processing for wound images. In order to make the wound area the main part of the image, we preprocess the wound image dataset to remove the background, and then feed it into the classifier for training. Therefore, we design a wound image localizer using YOLOv5 [50] to accurately detect and localize the relevant wound region in a wound image. Note that due to insufficient wound images, YOLOv5 is only used to localize the wound without classification. The images are compressed to 256 × 256 pixels and then data augmentation operations such as horizontal and vertical flipping are performed on each ROI image. Finally, 1536 images are acquired after augmenting 376 ROI images. In order to preserve the color characteristics of the wound, we do not modify the color of wound images to ensure that expanded images are similar to the original images. Additionally, all images are normalized. The ROI image dataset is divided into 70% training set, 20% validation set, and 10% test set to ensure that there is no overlap between the training and test sets.

Whole image processing of wound images. The training dataset has a total of 379 images with 8 images in each batch size. Each image in each batch is flipped by 15, 25, 35 and 45 degrees, respectively. The angle is limited to the range of 15–45 degrees in order to ensure that wound area is visible after rotation. Ultimately, the augmented training set has 1536 images. Similarly, images are uniformly compressed to 256 × 256 pixels, and are normalized to keep the similarity between the newly added images and the original images.

### 3.3. Training Details

We set the batch size to 8 and train all models for 300 epochs. A fully connected layer trained by a Rectified Linear Unit (ReLU) activation function with four hidden neurons is followed by a dropout layer with probability 0.3 to prevent overfitting. The dropout layer further reduces overfitting by randomly eliminating their contribution during training. We use the Adam optimizer with default $\beta_{1}$ = 0.9, $\beta_{2}$ = 0.999, with a learning rate of 0.0001. The goal of the optimization is to minimize the loss function by varying the model parameters. The NumPy and PyTorch libraries used in the models require the setup of random seeds. In order to ensure the reproducibility of our experiments, we set the random seeds to 3407. The number of wound samples in each category is more than 50, and the experiment finally classify the wounds into four categories: diabetic wounds, pressure wounds, surgical wounds and venous wounds. Finally, performance is evaluated using the trained models on test images, which are also scaled to 256 × 256 pixels during testing. We use Pycharm 2021.3.3 software (Community Edition), Python 3.7.16 programming language, and Pytorch 1.12.1 framework with CUDA 12.0 for GPU acceleration to train the model. In addition, we utilize torchvision 0.12.1 for data preprocessing, scikit-learn 1.0.2 for computing evaluation metrics, and matplotlib 3.5.3 for plotting. All experiments and model training were performed on a server equipped with GeForce RTX titan X. These software tools are all open-source.

### 3.4. Evaluation Metrics

The performance of deep learning models can be evaluated by using performance metrics such as Accuracy, Precision, Recall, and F1-score. Different evaluation metrics allows for a comprehensive comparison between models [51]. The performance of the model is expressed in the ratio of correct to incorrect classifications. The evaluation metric can be defined by a confusion matrix [27], where the correctly predicted positive and negative samples are denoted as True Positives (TP) and True Negatives (TN), and the incorrectly predicted positive and negative samples are denoted as False Positives (FP) and False Negatives (FN).

Accuracy indicates the percentage of correctly classified samples to the total samples, and is the most basic metric for evaluating the performance of a model. When the classes are balanced, the performance of the model can be measured accurately. The Accuracy is calculated as follows:

(7) $$Accuracy=\frac{TP+TN}{TP+FP+TN+FN}$$

Precision indicates the proportion of correctly classified positive samples among the actual positive samples in the prediction results. The expression for Precision is as follows:

(8) $$Precision=\frac{TP}{TP+FP}$$

The metric Recall quantifies the proportion of true positive samples that are correctly classified among the total predicted positive samples. The expression for Recall is given as follows:

(9) $$Recall=\frac{TP}{TP+FN}$$

The F1−score is computed by combining precision and recall measures. The expression is as follows:

(10) $$F1-score=\frac{2\times Precision\times Recall}{Precision+Recall}$$

The Receiver Operating Characteristic (ROC) Curve is adopted to evaluate the predictive capability of models. The key metrics in the ROC curve are True Positive Rate (TPR) and False Positive Rate (FPR), which are obtained by traversing through all thresholds to plot the entire curve.

True Positive Rate:

(11) $$TPR=\frac{TP}{TP+FN}$$

False Positive Rate:

(12) $$FPR=\frac{FP}{TN+FP}$$

## 4. Results

### 4.1. Ablation Experiment Analysis

Given that the classification of four wound types is the most complex and challenging task, we perform ablation experiments directly on the AZH dataset for the four-class classification. The purpose is to select the best combination of the two-branch network and clarify the effect of different modules for wound classification. The ablation experiment is designed as follows:

- HF-Net: In the first step, we use the models ResNet18, ResNet50, ResNet101, Res2Net50 and Res2Net101 pre-trained on the ImageNet dataset for four-class classification.
- LF-Net: Subsequently, only the MSDCRB is employed for four-class classification of wounds.
- HF-Net + LF-Net: Finally, the HF-Net responsible for extracting high-frequency information and the LF-Net responsible for extracting low-frequency information are combined for the four-class classification. Furthermore, Comparing the classification performance of AE-Res2Net50 + MSDCRB and AE-Res2Net101 + MSDCRB, which are obtained by adding attention and enhancement modules to HF-Net.

The results of the ablation experiments are shown in Table 3, including the Accuracy, Precision, Recall, and F1-score for all models. It is observed that all dual-branch models (HF-Net + LF-Net) outperform the models with only a single branch. Among them, the AE-Res2Net101+MSDCRB model exhibit the best performance, and we refer to this model as HLG-Net. The HLG-Net model achieves 82.61%, 79.10%, 75.80%, and 77.20% Accuracy, Precision, Recall, and F1-score for four-class classification of wound images, respectively. The ablation experimental results show that different models can cooperate to compensate for their respective shortcomings, and fusing image frequency information extracted from different modules can produce higher performance classifiers.

We utilize the MSDCRB as the fundamental module for LF-Net. Multiple MSDCRBs are employed to form a Multi-Stream Dilation Convolution Residual Group (MSDCRG). The number of groups and blocks has different effects on the wound classification task. The results of the four-class classification corresponding to different numbers of groups and blocks are shown in the Table 4. When Group = 3 and Block = 3, the highest classification accuracy can be achieved while maintaining a moderate number of parameters. Therefore, we adopt the configuration as our network module.

**Figure 4:**
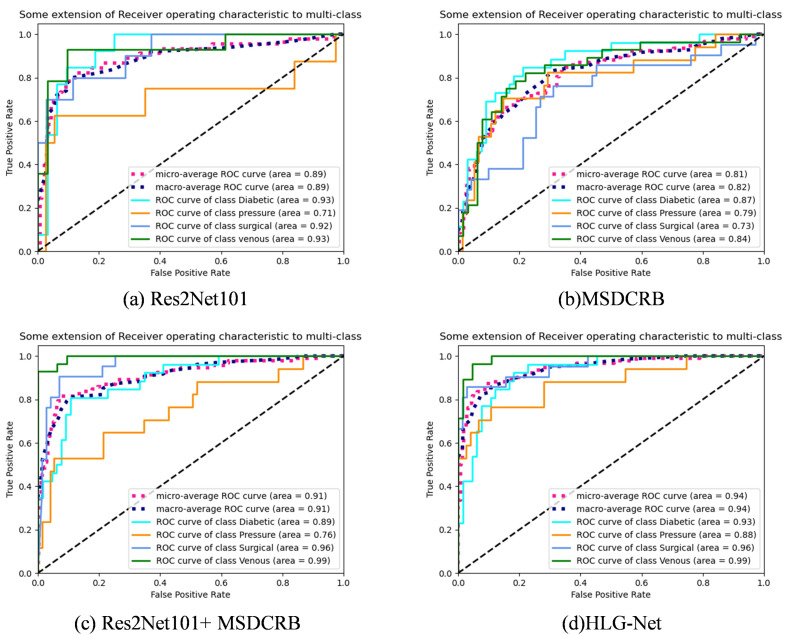
ROC curves of Res2Net101, MSDCRB, Res2Net101 + MSDCRB and HLG-Net models for four-class wound classification. (**a**) ROC curves of the Res2Net101 model for four-class classification. (**b**) ROC curves of the SDCRB model for four-class classification. (**c**) ROC curves of the Res2Net101 + MSDCRB model for four-class classification. (**d**) ROC curves of the HLG-Net model for four-class classification.

Figure 4 shows ROC curves of Res2Net101, MSDCRB, Res2Net101 + MSDCRB and HLG-Net models for four-class classification. The horizontal axis represents the FPR, while the vertical axis represents the TPR. It describes the connection between TP and FP. The macro-average ROC curve measures the macroscopic average ROC for four wound types. The micro-average ROC curve measures the microscopic average ROC for four wound types. The larger the Area Under Curve (AUC), the better the model. It can be observed that for the four wound types, the AUC of the Res2Net101 + MSDCRB model is greater than that of the Res2Net101 model or the MSDCRB model. The AUC of the AE-Res2Net101 + MSDCRB model is larger than Res2Net101 + MSDCRB model. The HLG-Net model achieves AUC values of 0.96 and 0.99 for Surgical, Venous classification, and 0.93 and 0.88 for Diabetic, Pressure classification. The model performs well in surgical and venous wound classification, followed by diabetic wounds, and relatively poorly in pressure wound classification. The reason for this result may be the limited data set and the significant variation between pressure ulcer images. The network may struggle to learn adequate features of pressure ulcers.

**Figure 5:**
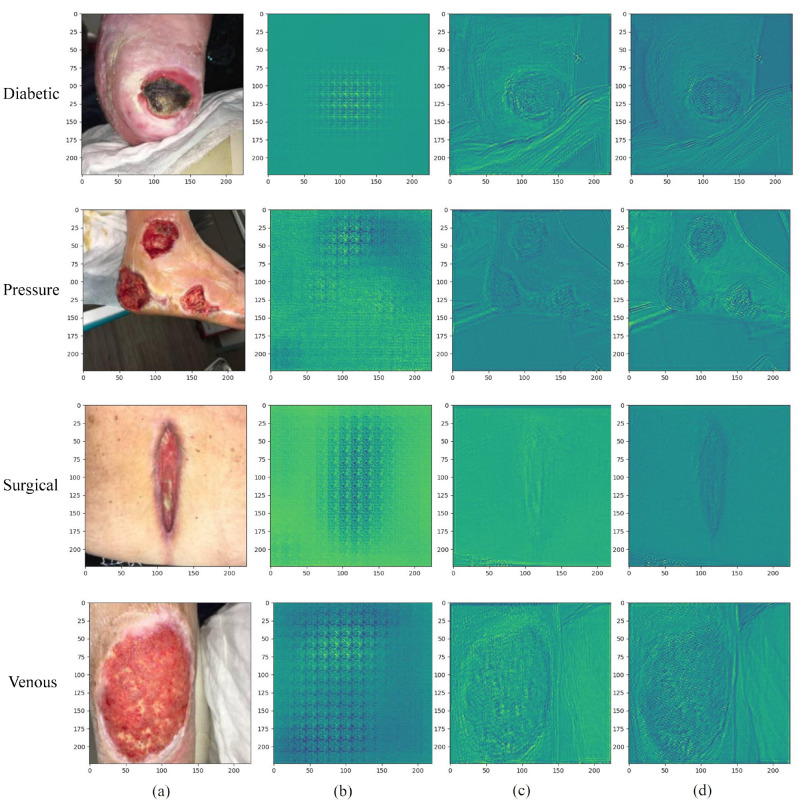
Visualization results of HF-Net, LF-Net and HLG-Net on diabetic foot ulcer wound image (first row), pressure ulcer wound image (second row), surgical wound image (third row) and venous ulcer wound image (fourth row). (**a**) Original image. (**b**) Feature map obtained from the HF-Net model. (**c**) Feature map obtained from the LF-Net model. (**d**) Feature map obtained from the HLG-Net model.

As in Figure 5, we show the results of feature map visualization obtained from different models, the first column is the original image of the wound. We directly visualize the feature maps of all channels without additional processing, and the output is the feature maps after multi-channel superposition. For comparison, we show the visualization results using HF-Net, LF-Net, and two-branch HLG-Net on the same image, respectively. As shown in Figure 5b, we can see from the visualization results that HF-Net mainly extracts the local detail of the wound, but neglects the low frequency information. In Figure 5c, LF-Net captures important features such as wound size, shape and location, but missing high-frequency information of the wound. In contrast, our proposed HLG-Net combines high-frequency and low-frequency information from both branches to capture more comprehensive features (shown in Figure 5d). The visualization results also reflect the classification performance.

**Figure 6:**
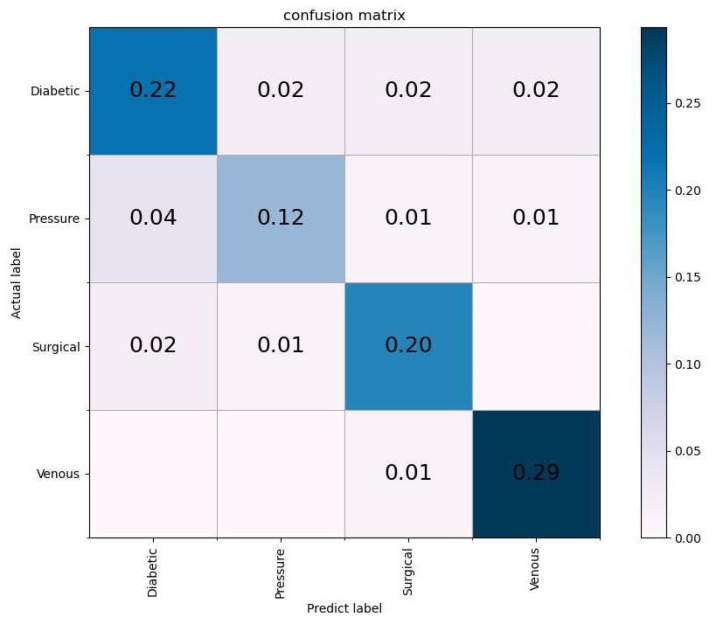
The confusion matrix of HLG-Net for four-class classification.

Based on the four-class classification confusion matrix of the HLG-Net model (shown in Figure 6), it can be observed that venous wounds have the lowest misclassification rate compared to other types of wounds. However, pressure wounds are more likely to be misclassified as diabetic wounds because of their similar visual appearance.

**Figure 7:**
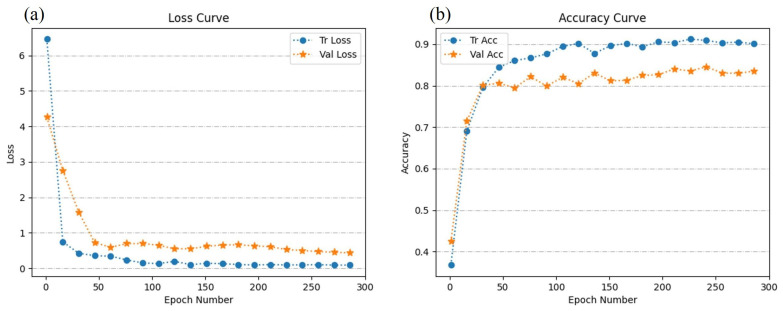
The learning curve of the HLG-Net model. (**a**) The loss curve of the HLG-Net model. (**b**) The accuracy curve of the HLG-Net model.

Moreover, during the training of the HLGNet model, we record the training loss, validation loss, training accuracy, and validation accuracy each 15 epochs. These data are used to construct loss curves and accuracy curves, as shown in Figure 7. The loss curve reflects the performance of the model on the training and validation sets, while the accuracy curve shows the corresponding classification accuracy. We observe that in the loss curve, the validation set loss is slightly higher than the training set loss, while in the accuracy curve, the validation set accuracy is slightly lower than the training set. The results indicate that the model performs relatively well on the training set, but is slightly overfitting on the validation set. This may be partly due to the small wound dataset, which causes the model to focus too much on the details and noise in the training data, affecting the generalization performance.

### 4.2. Hyperparameter Experimental Analysis

When deep learning models have a large number of parameters but are trained on insufficient wound image data, overfitting is a common concern. In our method, we employ data augmentation and dropout to mitigate overfitting. Data augmentation involves creating new images by altering existing images. Dropout refers to the process of randomly selecting a subset of neurons to be temporarily ignored during the forward propagation stage of model training, disabling their weights. These non-functional nodes are temporarily excluded from the network structure, but their weights are kept. In subsequent iterations, a new set of neurons is randomly hidden until training is complete. Since this process is stochastic, training a different network for each mini-batch enhances the generalization ability of the network. To prevent overfitting, we add a dropout layer after the fully connected layer. Table 5 presents the classification results for different dropout probabilities. The results indicate that incorporating dropout layers can effectively alleviate overfitting, and the best classification performance is achieved when the dropout probability is set to 30% in the network. Based on the results presented in Table 3, Table 4 and Table 5, we apply HLG-Net with a dropout level of 30% to two-class, three-class, and four-class classification tasks.

### 4.3. Classification Result Analysis

To evaluate and compare the performance of HLG-Net in classifying whole wound images and classifying wound ROI images, we perform experiments using two different datasets: one involving preprocessed whole wound images and another involving ROI images for training the classifier. The experimental results of binary classification for wound images are presented in Table 6.

**Figure 8:**
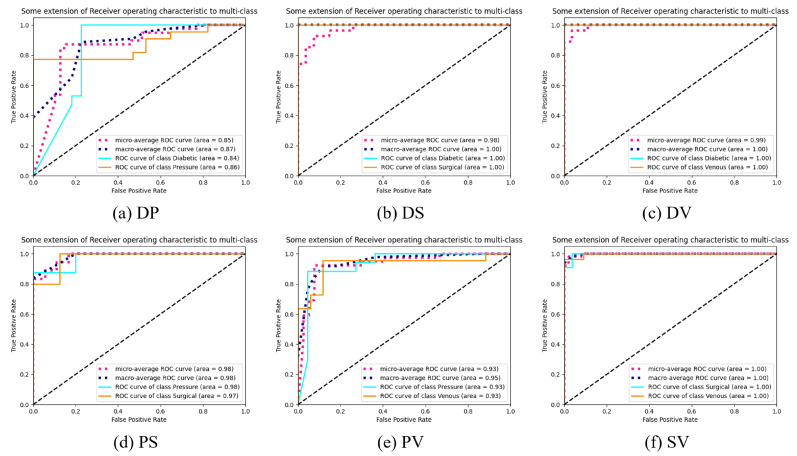
ROC curves of binary classification. (**a**) ROC curves of Diabetic, Pressure (DP) classification. (**b**) ROC curves of Diabetic, Surgical (DS) classification. (**c**) ROC curves of Diabetic, Venous (DV) classification. (**d**) ROC curves of Pressure, Surgical (PS) classification. (**e**) ROC curves of Pressure, Venous (PV) classification. (**f**) ROC curves of Surgical, Venous (SV) classification.

We conduct six binary classifications on the AZH dataset, namely DP, DS, DV, PS, PV, and SV. Table 6 presents the specific results of binary classifications. It can be seen that among all the binary classifications, HLG-Net performs better in classifying the whole wound image compared to classifying the ROI images. The HLG-Net model performs well in distinguishing between surgical wounds and venous wounds, achieving 98.00%, 95.70%, 100%, and 97.80% for Accuracy, Precision, Recall, and F1-score, respectively. The DP classification is the lowest accuracy, and distinguishing diabetic wounds from pressure wounds is the most challenging task. As shown in Figure 8, the ROC curves for the binary classification of the whole wound images show that HLG-Net has AUC values close to 1 for DS binary classification, DV binary classification, PS binary classification and SV binary classification. These results indicate that HLG-Net performs well in the binary classification of wounds. However, the classification performance is relatively poor for the DP binary classification, with AUC values of 0.84 and 0.86, respectively.

The three-class classification results are shown in Table 7. For the DSV, the network achieves Accuracy of 92.11%, Precision of 89.80%, Recall of 90.00%, and F1-score of 89.60%. As for the PSV classification, the Accuracy, Precision, Recall, and F1-score are 88.06%, 84.70%, 80.10%, and 80.80%, respectively. However, the classification performance for the DPS and DPV is relatively poor. For DPS, the Accuracy, Precision, Recall, and F1-score are 74.19%, 69.90%, 79.80%, and 74.20%, respectively. For DPV, the Accuracy, Precision, Recall, and F1-score are 77.14%, 69.40%, 69.70%, and 69.40%, respectively. Similarly, in all three-class classification, HLG-Net shows superior performance for classifying whole wound images compared to classifying ROI images. Classifying diabetic wounds, pressure wounds and surgical wounds is the most challenging task.

**Figure 9:**
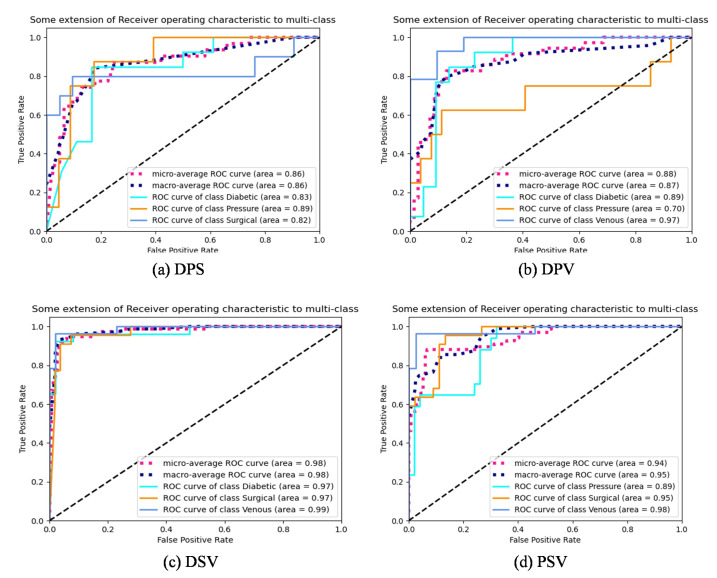
ROC curves of three-class classification. (**a**) ROC curves of Diabetic, Pressure, Surgical (DPS) classification. (**b**) ROC curves of Diabetic, Pressure, Venous (DPV) classification. (**c**) ROC curves of Diabetic, Surgical, Venous (DSV) classification. (**d**) ROC curves of Pressure, Surgical, Venous (PSV) classification.

As shown in Figure 9, HLG-Net achieves AUC values above 0.97 for the three-class classification of diabetic, surgical, and venous wounds, indicating the best classification performance. However, for the classification of diabetic, pressure, and venous wounds, the performance is comparatively poorer, with AUC values of 0.89, 0.70, and 0.97, respectively. The classification performance is least effective for the three-class classification of diabetic, pressure, and surgical wounds, with AUC values of 0.83, 0.89, and 0.82, respectively. Among the pressure, surgical, and venous wound classification, both Surgical and Venous classes achieve AUC values of 0.95 or higher, while the Pressure class has a comparatively lower AUC value of 0.89.

For the most complex four-class classification of wound images, the HLG-Net model achieves an accuracy, precision, recall and F1-score of 82.61%, 79.10%, 75.80% and 77.20%. Table 8 presents results for the four-class classification, indicating that the overall performance of classifying whole wound images is superior to classifying ROI images.

## 5. Discussion

### 5.1. Comparison of HLG-Net Model for Different Classification Tasks

**Figure 10:**
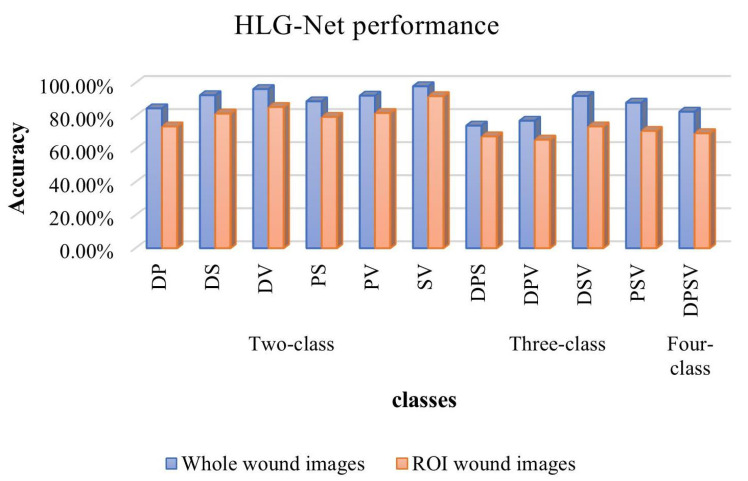
Performance comparison of HLG-Net on the mixed classification of whole wound images and ROI wound images.

Figure 10 presents a comprehensive comparison of all classification results using whole wound images and ROI wound images. It is evident that the proposed method outperforms the classification performance when using whole wound images compared to ROI wound images. This result may be due to the whole image usually containing information about the body part where the wound is located. For example, diabetic wounds mainly occur on the feet, while venous wounds are usually located on the legs. In addition, whole images can provide contrast between the wound and its surrounding tissue, allowing the model to better distinguish the difference between the wound and surrounding normal tissue. For some ulcers, such as pressure ulcers, shape and size may be important characteristics. The multi-scale features learned from whole images may be able to improve the generalization of the model for different wound types. Overall, the whole image provides more comprehensive and abundant information than the ROI image, which is important for the classification of diabetic foot ulcers, pressure ulcers, venous ulcers, and surgical wounds. In this case, the model is able to understand the wound as a whole and its surrounding environment, thereby improving classification performance. Many previous models require extensive preprocessing, lesion segmentation, and extraction of wound regions before classification. In contrast, the HLG-Net model does not require excessive processing and can directly classify whole wound images.

**Figure 11:**
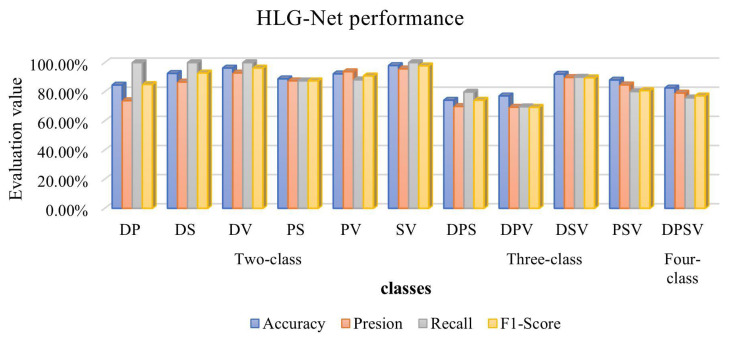
Performance comparison of HLG-Net on AZH dataset for mixed classification.

We conduct a four-class classification, four three-class classifications, and six two-class classifications. In the four-class classification, the highest accuracy is 82.61%. Among the three-class classifications, the highest accuracy is obtained in the classification of DSV, reaching 92.11%. Among the two-class classifications, the highest accuracy is achieved in the classification of SV, with an accuracy of 98.00%. From the comparison of all the results in Figure 11, it can be observed that the classification performance improves when the number of classes from 4 to 3 and 2. This is because from four classifications to three classifications and then to two classifications, the tasks that the model needs to handle gradually become simpler. In simpler tasks, the model may more easily converge to a better state. In contrast, in complex tasks, the model may take longer to converge and may even get stuck in a local minimum. In addition, as the task becomes progressively simpler, the distinction between classes may increase. The model learns features more easily which can clearly distinguish each class, so performance gradually increases. In conclusion, these experimental results demonstrate the reliability of HLG-Net for wound classification.

### 5.2. Comparison with Previous Works

We only compare our work with studies that include the wound types in our experimental dataset. Due to the scarcity of wound datasets, most related articles mainly focus on binary classification problems, such as distinguishing normal samples from abnormal samples. In our experiments, we do not include normal skin and background images. Therefore, we do not make the comparison here.

In comparison to the study conducted by Jens et al. on the classification of D and V [52], we employ a smaller dataset for D and V classification. Our method achieves 1.3% lower precision than previous work, but a recall of 100%. We conduct experiments using the AZH dataset, similar to the study conducted by Anisuzzaman et al. [30]. In comparison to their study, we conduct 11 similar experiments, with 3 experiments having slightly lower accuracy and 8 experiments having significantly higher accuracy. The eight experiments with higher classification accuracy are as follows. The classification accuracy increased by 3.26% for DPSV, 1.44% for DSV, 1.83% for PSV, 1.24% for DPS, 3.95% for DS, 3.71% for DV, 7.31% for PS, and 2.73% for PV. The three experiments with relatively low classification accuracy are as follows. The classification accuracy is reduced by 3.85% for DPV, 0.38% for DP, and 0.08% for SV. We have two similar experiments with Rostami et al. [29]. Specifically, the accuracy of classifying DSV increases by 0.21%, while the accuracy of classifying SV is 1.6% higher. Note that this comparison may not be entirely fair due to difference in dataset sizes and segmentation proportions.

HLG-Net has significant advantages over other works in meeting current needs. The specific advantages are as follows.

- Compared with existing literature, we use a high- and low-frequency dual-branch classification network. This structure contributes a new perspective. Through the high and low frequency information of the wound image, we are able to more comprehensively capture the characteristics of the input wound image. Table 9 demonstrates that HLG-Net significantly improves the classification performance.
- In the experiments, HLG-Net deliberately excludes normal skin and background images, which is different from other works that usually focus on binary classification problems containing normal and abnormal samples. This exclusion allows HLG-Net to focus on handling the most complex classification tasks involving only wounds, which is more suitable for practical medical applications.
- The method of Anisuzzaman et al. [30] involves using wound images and the location of the wound, and requires different models in different tasks to achieve the highest classification accuracy. In contrast, HLG-Net can achieve relatively good results for multiple mixed classes using only wound images and a single model, which is more consistent with practical applications.
- Compared with other models that require complex data preprocessing in wound image classification tasks, HLG-Net shows good performance by directly using the whole wound image for training. The simplified data processing method makes our model more versatile and applicable, reducing complex pre-processing steps. Therefore, HLG-Net effectively meets the current demand for a more accurate and versatile wound classification method.

However, our work also has certain limitations compared with other works. When the difference in high- and low- frequency information of the wound image is too small, the feature extraction part of the network may not have enough complexity to capture these subtle differences. And the dual-branch network model has many parameters, which results in long training time.

## 6. Conclusions

CNN-based methods often struggle to learn sufficient features from wound images when the data is limited. To address the complexity of different wound images and the overfitting issue associated with small-scale datasets, we propose the HLG-Net model, which can simultaneously learn high and low-frequency information of wound images for multi-class wound classification. By concatenating and combining the high and low-frequency features, a more comprehensive understanding of wound image features can be obtained. The HLG-Net model achieves superior performance compared with all single-branch and other two-branch models, almost outperforming previous experimental methods. Table 8 demonstrates that the proposed method achieves an Accuracy, Precision, Recall, and F1-score of 82.61%, 79.10%, 75.80%, and 77.20% for the four-class classification of wounds, respectively. To better understand the deep learning model, we also visualize the feature maps of the training model, revealing the role of different branches. In conclusion, HLG-Net can effectively utilize high and low frequency features of wound images, thus improving classification performance. Extensive experiments are conducted on the AZH dataset using both the whole wound images and ROI wound images for two-class, three-class and four-class wound classifications. Regardless of the classification task, our model shows better performance on the whole wound images. The whole wound images require only simple data preprocessing, making our model more general and applicable.

Limitations. Although our HLG-Net achieves encouraging performance, it still has the following limitations. Firstly, the work lacks diverse sample datasets, resulting in the generalization ability of the model in general. Secondly, the dual-branch network leads to too many model parameters, increases the complexity of the network, and slows down the training and inference speed of the model.

Future Solutions. To further improve model performance and generalization capabilities, we can try to establish a standardized public dataset in the future work. By collaborating with medical institutions to capture images containing different wound types, stages of treatment, and different medical environments. At the same time, it is important to strengthen cooperation with ethical experts to protect patient privacy. Considering the problem of numerous model parameters and long training time, we are able to further introduce model compression technology, such as model pruning, knowledge distillation and quantization. These methods can reduce the size of the model while maintaining or improving model performance. Moreover, we can use hardware accelerators for decentralized training and reduce training time. In conclusion, the performance of wound classification models can be continuously optimized to fit the needs of the medical image processing field.

## Figures and Tables

**Table 1:** Class label abbreviations.

Abbreviation	Description
Diabetic wounds	D
Pressure wounds	P
Surgical wounds	S
Venous wounds	V

**Table 2:** AZH dataset splitting.

Class	Training	Validation	Test	Total
Diabetic	108	31	15	154
Pressure	71	19	10	100
Surgical	90	26	12	128
Venous	110	31	15	156

**Table 3:** The Accuracy, Precision, Recall, and F1-score of four-class wound classification for each model.

Network		Accuracy	Precision	Recall	F1-Score
HF-Net	ResNet18	68.48%	62.20%	61.60%	61.80%
	ResNet50	71.74%	67.30%	57.70%	61.40%
	ResNet101	73.91%	71.90%	66.80%	68.50%
	Res2Net50	72.83%	68.70%	60.50%	63.10%
	Res2Net101	75.56%	78.70%	69.00%	71.70%
LF-Net	MSDCRB	60.87%	54.10%	48.20%	50.10%
HF-Net + LF-Net	ResNet18 + MSDCRB	70.65%	67.60%	57.10%	61.60%
	ResNet50 + MSDCRB	72.83%	75.80%	61.30%	67.60%
	ResNet101 + MSDCRB	76.09%	80.00%	63.20%	69.40%
	Res2Net50 + MSDCRB	78.26%	81.60%	67.60%	71.80%
	Res2Net101 + MSDCRB	80.43%	74.90%	75.70%	74.60%
	AE-Res2Net50 + MSDCRB	79.35%	80.10%	70.60%	75.00%
	**HLG-Net**	**82.61%**	**79.10%**	**75.80%**	**77.20%**

**Table 4:** The Accuracy, Precision, Recall, and F1-score obtained from different numbers of MSDCRG and MSDCRB.

(Group,Block)	Accuracy	Precision	Recall	F1-Score
(1,2)	73.33%	71.50%	60.70%	64.20%
(2,2)	77.17%	75.90%	68.50%	71.70%
(2,3)	81.52%	76.10%	78.70%	76.80%
(3,2)	79.35%	73.40%	70.50%	71.00%
**(3,3)**	**82.61%**	**79.10%**	**75.80%**	**77.20%**
(4,3)	79.35%	73.40%	70.50%	71.00%
(4,4)	77.17%	75.90%	68.50%	71.70%

**Table 5:** The Accuracy, Precision, Recall, and F1-score of different dropout level.

Dropout	Accuracy	Precision	Recall	F1-Score
0	76.09%	76.90%	64.00%	66.00%
10%	77.78%	75.30%	69.00%	71.00%
20%	81.52%	77.90%	74.50%	76.10%
**30%**	**82.61%**	**79.10%**	**75.80%**	**77.20%**
40%	81.52%	77.90%	74.50%	76.10%

**Table 6:** The Accuracy, Precision, Recall, F1-score of binary classifications.

Binary		Accuracy	Precision	Recall	F1-Score
Whole images	DP	84.62%	73.90%	100%	85.00%
	DS	92.59%	86.70%	100%	92.90%
	DV	96.30%	92.90%	100%	96.30%
	PS	88.89%	87.50%	87.50%	87.50%
	PV	92.31%	93.80%	88.20%	90.90%
	**SV**	**98.00%**	**95.70%**	**100%**	**97.80%**
ROI images	DP	73.68%	63.60%	87.50%	73.70%
	DS	81.48%	100%	61.50%	76.20%
	DV	85.45%	95.00%	73.10%	82.60%
	PS	79.49%	71.40%	88.20%	78.90%
	PV	81.82%	70.00%	87.50%	77.80%
	SV	92.00%	90.90%	90.90%	90.90%

**Table 7:** The Accuracy, Precision, Recall, F1-score of three-class classifications.

Three-Class		Accuracy	Precision	Recall	F1-Score
Whole images	DPS	74.19%	69.90%	79.80%	74.20%
	DPV	77.14%	69.40%	69.70%	69.40%
	**DSV**	**92.11%**	**89.80%**	**90.00%**	**89.60%**
	PSV	88.06%	84.70%	80.10%	80.80%
ROI images	DPS	67.69%	54.40%	58.10%	56.20%
	DPV	65.71%	75.00%	69.20%	61.10%
	DSV	73.68%	77.50%	58.00%	66.40%
	PSV	70.97%	69.70%	70.40%	70.97%

**Table 8:** The Accuracy, Precision, Recall, F1-score of four-class classifications.

Four-Class		Accuracy	Precision	Recall	F1-Score
Whole images	**DPSV**	**82.61%**	**79.10%**	**75.80%**	**77.20%**
ROI images	DPSV	69.57%	64.50%	61.90%	62.00%

**Table 9:** A comparison of previous works and present work.

Classes	Evaluation Metrics	References	Previous			Present	
			Models	Dataset	Result	Model	Result
DPSV	Accuracy	Anisuzzaman et al. [30]	VGG16 + LSTM	AZH	79.35%	HLG-Net	**82.61%**
DSV	Accuracy		VGG19 + MLP		90.67%		**92.11%**
PSV	Accuracy		VGG16 + MLP		86.23%		**88.06%**
DPS	Accuracy		VGG16 + LSTM		72.95%		**74.19%**
DPV	Accuracy		VGG19 + MLP		**80.99%**		77.14%
DP	Accuracy		VGG16 + MLP		**85.00%**		84.62%
DS	Accuracy		VGG16 + MLP		88.64%		**92.59%**
DV	Accuracy		VGG16 + MLP		92.59%		**96.30%**
PS	Accuracy		VGG16 + MLP		81.58%		**88.89%**
PV	Accuracy		VGG16 + MLP VGG19 + MLP		89.58%		**92.31%**
SV	Accuracy		VGG19 + MLP		**98.08%**		98.00%
DSV	Accuracy	Rostami et al. [29]	Ensemble DCNN- based classifier	AZH	91.90%		**92.11%**
SV	Accuracy				96.40%		**98.00%**
DV	Precision Recall	Jens et al. [52]	YoloV5m6	435 diabetic foot ulcers and 450 venous leg ulcers	**94.20%** 83.70		92.90% **100%**

## Data Availability

Data are not available at present because there are further research plans. If necessary, you can contact the author to obtain the data.
